# Physical Activity in The Therapy of Overweight and Obesity in Children and Adolescents. Needs and Recommendations for Intervention Programs

**DOI:** 10.34763/devperiodmed.20172103.224234

**Published:** 2017-10-28

**Authors:** Wiesław Osiński, Adam Kantanista

**Affiliations:** 1 Department of Physical Activity Science and Health Promotion, Poznan University of Physical Education, Poznan Poland; 2 Department of Didactics of Physical Activity, Poznan University of Physical Education, Poznan Poland

**Keywords:** exercise, aerobic training, anaerobic training, resistance training, youth, ćwiczenia fizyczne, trening aerobowy, trening anaerobowy, trening oporowy, młodzież

## Abstract

Overweight and obesity may lead to serious health problems, and negatively affect everyday functioning in physical, psychological and social spheres. The high prevalence of overweight and obesity in children and adolescents constitutes a huge public health burden. One way of designing and implementing behavioral interventions aimed at the reduction of adipose tissue is to promote physical activity. In this review we present recommendations regarding the planning, monitoring and implementation of intervention programs involving controlled physical activity. Considering specific individual determinants and needs in terms of improving children’s health, physical condition and physical performance, we have separately discussed recommendations for interventions involving aerobic and anaerobic exercises with moderate and high intensity, including high-intensity interval training (HIIT). We have also focused on the role of resistance training. Finally, we have emphasized that children and adolescents with overweight or obesity should also be motivated to undertake activities referred to as non-exercise activity thermogenesis (NEAT).

## Streszczenie

Nadwaga i otyłość wiążą się z bardzo poważnymi problemami zdrowotnymi oraz znajdują swoje negatywne konsekwencje w sferach codziennego fizycznego, psychicznego i społecznego funkcjonowania jednostki. Powszechne występowanie nadwagi i otyłości wśród dzieci i młodzieży stanowi jeden z najpoważniejszych problemów zdrowia publicznego. W projektowaniu i przeprowadzaniu interwencji behawioralnych ukierunkowanych na redukcję tkanki tłuszczowej poszukuje się skutecznych rozwiązań również w zakresie zwiększenia poziomu aktywności fizycznej. W pracy zaprezentowano zalecenia dotyczące planowania, monitorowania i realizacji programów interwencyjnych z wykorzystaniem sterowanej aktywności fizycznej. Mając na uwadze specyficzne indywidualne uwarunkowania i potrzeby w zakresie poprawy zdrowia, kondycji i sprawności fizycznej omówiono zalecenia dotyczące interwencji, w których stosuje się aerobowe oraz anaerobowe wysiłki o umiarkowanej i wysokiej intensywności, a w tym również high-intensity interval training (HIIT). Odrębnie scharakteryzowano rolę treningu oporowego. Zwrócono uwagę na potrzebę zachęcania do podejmowania przez dzieci i młodzież z nadwagą i otyłością aktywności określanej jako non exercise activity thermogenesis (NEAT).

## Introduction

Overweight and obesity not only represent a serious health problem, but may also negatively affect one’s physical performance, functioning in esthetic, psychological and economic spheres, as well as social functioning and life in general. Children with overweight and obesity frequently suffer from an inferiority complex, experience rejection by their peers, and have problems with social contacts. They believe that other children laugh at them, want to draw others’ attention at any cost, and generally tend to underachieve [1, 2].

The diagnosis and therapy of obesity may be associated with many complications, changes in therapeutic responses, and dramatic deterioration of prognosis. Designing and implementing behavioral intervention requires the identification of effective solutions at both the environmental and individual level. Overweight and obesity in children and adolescents constitute a serious public health burden.

Pooled data for Western countries suggest that approximately 50% of adults and 10-20% of children and adolescents are overweight or obese [3]. Concerns have been raised that this may slow down the growth tendency in life expectancy that has been observed in various populations for many decades. A longitudinal study conducted in 2010 and 2013 by researchers from the National Food and Nutrition Institute in Warsaw [4] demonstrated that prevalence of overweight and obesity in 11- to 12-year-old boys increased from 23.7% to 28.0%, whereas the percentage of obese girls from the same age group remained essentially similar, 22.8% and 22.0%, respectively. Comparative analysis of these results and data for similarly aged children from other European countries demonstrated that prevalence rates for overweight and obesity among Polish youth start resembling those reported in countries whose citizens evidently suffer from excess body weight [3]. Another representative study systematically conducted by researchers from the National Food and Nutrition Institute showed that the percentage of children with overweight or obesity doubled over a 35-year period between 1971 and 2006. The rapid increase in the prevalence of overweight and obesity among children and adolescents is particularly alarming. Research showed that in 70% of the persons who become obese at a young age, obesity also persists later in life [5].

The aim of the present work is to provide a concise theoretical and practical overview of the latest information on recommendations for implementing the intervention programs among overweight and obese children and adolescents, based on different types of exercise.

## Rationale and general guidelines for physical activity programs aimed at therapy of overweight and obesity

### Requirements regarding the reduction of body weight with simultaneous improvement of health, physical condition and physical performance

Major international organizations share the same or at least quite similar opinions regarding recommended levels of physical activity. However, it should be emphasized that these are general guidelines for prevention of cardiovascular diseases, diabetes mellitus, obesity, osteoporosis and other conditions. From a medical perspective, the recommended level of physical activity in children and adolescents (5 to 17 years of age) was defined as at least 60 min of moderate to intense physical exercise a day, under the assumption that higher levels of physical activity may produce additional health benefits. Physical activity should be based on aerobic exercises. Intense training should be undertaken three times a week, and include exercises that promote muscle strength and stimulate the skeletal system [6, 7].

Searching for an efficient solution to the emerging problem of excess body weight in Poland, specialists from the National Food and Nutrition Institute have developed the “Nationwide Program to Control Overweight and Obesity within the Healthcare System” [4]. Patients enrolled in this program are to be provided with complex care offered by a physician, dietician, psychologist and rehabilitation specialist. Each edition of the program should last at least 12 weeks and include: 1) rehabilitation (at a swimming pool or gym) 2-3 times per week; the exercises should be stratified according to intensity and be suitable for subjects in various general physical condition; 2) dietary counseling in the form of individual consultations and group workshops; and 3) group meetings with a psychologist, aimed at both education and therapy.

During the first stage of enrollment in the program, candidates are subjected to complex biochemical tests (lipidogram, uric acid concentration, complete blood count, transaminase activity, oral glucose tolerance test, TSH level). Furthermore, they need to fulfill the strict inclusion and exclusion criteria of the program. Persons participating in the program also have unlimited access to dietary counseling at dedicated consultation units [4]. However, children and adolescents with overweight or obesity who suffer from various comorbidities and frequently need to take multiple medications, as well as persons with disabilities, require a more specific approach.

A program aimed at the reduction of body weight will be most effective if dietary restrictions are combined with physical training and enforcement of other health behaviors. Systematic physical training is vital both for the initial reduction of body weight and for its further maintenance at a target level. Training produces calorie deficits due to the thermal effect of physical activity and due to an increase in the metabolic rate resulting from muscle mass gain or at least its maintenance at the baseline level.

Children and adolescents who are satisfied with physical training are more likely to participate in the program than those who had solely been recommended a restrictive diet. Furthermore, such an approach is markedly more effective and poses a lesser health threat. The dietary principles and the rules of body weight control that need to be followed by participants of such a program are specified below.

1. Adequate diet should contain appropriate proportions of protein, carbohydrates, fats, vitamins, minerals and water. The subjects should comply with the recommended number of daily meals defined by a certified dietician.
2. Body weight loss should result from a decrease in fat mass, rather than from a reduction of lean (muscle) mass.
3. Body weight should not be reduced too rapidly; usually the weekly decrease in body weight should not be greater than 0.5-0.8%.
4. All pills, either enhancing metabolic rate or reducing appetite, should be avoided. Pharmacotherapy can be used solely periodically, under the restrictive control of a physician and according to his/ her prescription.
5. The rate of excess body weight reduction should be gradually decreased in consecutive weeks, but an adequate (not too restrictive) diet should still be maintained, along with an elevated level of physical activity and a healthy lifestyle [8, 9, 10].

A well-designed body weight reduction program should have a predefined structure, with clearly specified objectives, adjustment to all factors identified during individual and environmental diagnosis, and a prognosis based on rational premises. Individualized dietary guidelines and recommendations regarding the form, intensity, duration and frequency of physical activity should be documented in detail. The program of physical training should be adjusted to the capabilities and preferences of children and adolescents, with a gradual increase in intensity and duration. Exercises included in the program should cover as many components of health-related fitness (H-RF) as possible, i.e. focus on morphological, muscular, motor, cardiorespiratory and metabolic parameters [8, 11].

In some cases the implementation of a physical activity program may be highly challenging. This refers to patients with severe obesity, a long-term history of a sedentary lifestyle, disability or chronic disease. In such cases physical exercises should be considered a component of clinical intervention and therefore should be supervised by appropriately trained physiotherapists, under a physician’s control. Children and adolescents with severe obesity are particularly prone to premature atherosclerosis, arterial calcification, deposition of “bad” cholesterol and loss of vascular elasticity. Due to progressive degeneration, their myocardium may be deprived of oxygen and nutrients. Destructive processes may also lead to a decrease in the vital capacity of the lungs, worse gas diffusion, limited mobility of the chest and lesser elasticity of pulmonary tissue [12, 13].

Children and adolescents with obesity may also present with enhanced degenerative changes in the skeletal and articular system, with premature loss of osteoblast function and growth of cancellous bone, preterm demineralization and a resultant increase in fracture risk. Weakening of the ligamentous-capsular system and its exposure to additional strain are reflected by a substantial increase in injury risk. Reduced contents of calcium and potassium impair the physiological contracture of the muscles [14]. All these unfavorable processes occurring at the early stages of ontogenesis are additionally enhanced due to refraining from systematic participation in physical education programs, usually observed in overweight or obese subjects [15].

### The role of non-exercise activity thermogenesis (NEAT)

Overall energy balance is also affected by energy expenditure during many activities of daily living that are not directly related to sport or intentional exercise, such as climbing down the stairs instead of using an elevator, and longer walks, e.g. going to school on foot, participation in organized motor games and activities, riding a bike, going for a trip, etc. The energy burned while doing this type of physical activity is referred to as NEAT. The level of NEAT in a given subject is determined by biological factors, such as sex, body weight, body composition and age, as well as by environmental factors. While variance in NEAT may be an occasional, not otherwise programmed phenomenon, it sometimes represents a characteristic personality trait or a measure of one’s interests and aspirations in life, significantly affecting the person’s energy balance and contributing to changes in body composition [16].

Balance between energy expenditure and dietary intake of calories is a key determinant of stable body weight, overweight, obesity or underweight. Energy balance consists of three basic components: a) basal metabolic rate, b) thermal effect of food, and c) thermogenesis. Thermogenesis can be associated with physical activity or NEAT. In a large proportion of children and adolescents, the former type of thermogenesis is substantially reduced or virtually absent. Under such circumstances most energy expenditure results from NEAT associated with all forms of spontaneous activity, including involvement in recreational games, various forms of locomotion, household and garden activities, shopping, etc. [16].

NEAT shows considerable intrapersonal and interpersonal variability. The contribution of NEAT to daily energy expenditure is person-specific, ranging from 15% to 50% and more depending on the level and type of one’s daily life activities. Also genes were shown to contribute to the individual variability in NEAT [17]. Evidence from twin and family studies suggests that the heritability of physical activity ranges from 29% up to 62% [16, 18].

Technical progress frequently contributes to a substantial decrease in NEAT, which is not counterbalanced by greater energy expenditure during intentional physical activity. The proportion of children who go to school on foot is still decreasing, similarly to the percentage of adults who walk to their workplace instead of commuting; furthermore, most of the latter have sedentary work. As a result, the NEAT of adults with similar anthropometric characteristics may differ by up to 2000 kcal/day. NEAT in obese subjects was shown to be 2.5 h/ day lower than in persons with normal body weight who neither do sports nor undertake recreational physical activity. Persons who were not obese, significantly more often went for a walk, used stairs instead of an elevator and were involved in numerous household activities [16].

According to the literature, NEAT may be controlled by: a) central mediators, b) hormones, and c) peripheral signaling. One example of such an association is the release of neuromediators that regulate hunger and appetite. Previous studies dealing with the problem in question analyzed the associations of NEAT with concentrations of hormones, leptin and thyroxin [19, 20]. Also the sympathetic nervous system may modulate the level of spontaneous physical activity and NEAT. However, the contribution of many peripheral signals, such as hunger and fatigue, and their role as determinants of physical activity are still incompletely understood.

### Enrollment and consent to participate in the program

Prior to the enrollment in a physical activity program aimed at reducing body weight, the health status of potential participants needs to be determined, along with their physical condition, and if applicable, also their disability level. A prerequisite for safe participation in the program is the evidence of a recent medical checkup. A key issue is to exclude all the absolute contraindications to physical exercise that might preclude participation in the program or postpone enrollment until the possible improvement of the candidate’s health status [21, 22, 23]. Children and adolescents with severe diseases and ailments (frequently resulting from obesity) present with markedly reduced functional performance and mobility, and prior to the enrollment, should undergo a complex medical examination (by a family physician, endocrinologist, gastrologist, cardiologist and other specialists whenever applicable), physical performance and functional fitness tests, evaluation by a dietician and psychotherapist.

Children and adolescents diagnosed with heart failure, ischemic heart disease, resistant/ refractory hypertension, diabetes mellitus requiring pharmacotherapy, uncontrolled hypothyroidism or hyperthyroidism, epilepsy, and disability which interferes with exercise capability, should participate in a weight reduction program under the constant supervision of a physician.

An important element in the preliminary enrollment in the program is an informed consent form. The form should contain the basic information about the institution (center) to which the program is affiliated, its mission, principal initiatives, mutual responsibilities and obligations. In the case of children and adolescents, the form needs to be signed by their parents or legal guardians. The data included in the form are analyzed by a physician and physical activity instructor, and archived at the affiliated center. Prior to the enrollment, information about all the medications that may potentially limit participation in physical training or change the bodily response to physical exercise needs to be obtained, along with the dosing information.

## The role of moderate- and high-intensity physical exercise and resistance training in the therapy of overweight and obesity

### Fat-burning zone

Physical exercise should improve the efficiency of metabolic processes, promote the effective reduction of adipose tissue and result in the maintenance or even increase of muscle mass. Mechanisms that protect against the loss of lean body mass during aerobic or resistance training include the increased activity of growth hormone, adrenalin and noradrenalin. These hormones upregulate lipases, i.e. enzymes that catalyze the degradation of fat to glycerol and fatty acids. The latter are metabolized and serve as a source of energy during aerobic exercise. The key hormones involved in resistance training are testosterone and growth hormone that stimulate the synthesis of protein and thus contribute to the development of muscle mass [24, 25].

Training can be considered effective if it is based on exercises that directly activate the cardiorespiratory function due to the rhythmic involvement of large muscle groups over a possibly long period of time. However, the training program should not be based solely on the strict assumptions derived from experimental and biochemical-functional studies, but should also be adjusted to individual preferences and the psychosocial context.

The question concerning what exercise intensity produces the most desirable effects is still a matter of discussion. According to Romijn et al. [26], the most effective reduction of body fat is observed at exercise intensity corresponding to 65% of VO_2_max. Further increase in exercise intensity, beyond the so-called fat-burning zone (85% VO_2_max), results in less effective reduction of adipose tissue content. These findings contributed to the assumption that exercises with lesser intensity (such as marching, running, riding a bike, rowing, etc.) but longer duration are the most effective way to reduce body fat. However, in recent years, this statement has been increasingly put into question [27, 28]. Although researchers generally agree that greater intensity of training results in a relative decrease in the adipose tissue reduction rate in favor of the degradation of other components, e.g. carbohydrates, total energy expenditure is still greater than during moderate-intensity exercise. This implies that more intense exercise may not only contribute to effective reduction of body fat, but may also enhance post-training metabolism. However, high-intensity exercises may pose a threat for many persons with overweight and obesity and therefore should be implemented with caution.

### Post-exercise increase in resting metabolic rate

Total energy expenditure depends on resting metabolic rate (RMR) and additional expenditure during physical activity. RMR corresponds to energy expenditure required for the maintenance of vital functions at rest. The resting metabolic rate is sex-specific and determined by the proportion of muscles to adipose tissue. Since adipose tissue is less metabolically active, persons with more muscular bodies (with greater content of muscle tissue) are generally characterized by higher RMR than individuals with body adiposity [29].

Increased energy expenditure is also observed post-exercise, during recovery after intensive training. The restitution period starts with the repair of minor muscle injuries, followed by the elimination of unnecessary metabolites and repletion of energetic compounds. These processes require additional energy expenditure, therefore the metabolic rate remains enhanced despite discontinuation of physical exercise. Depending on the physical activity level, post-training energy expenditure may represent up to 60-70% of overall energy consumption [30, 31, 32]. Moderate and intense aerobic exercise leads to an increase in the post-training resting metabolic rate from 5% to 16%, which may persist for 12 to 39 hours [8, 32].

### Recommendations regarding aerobic exercises, programming aerobic training and monitoring its intensity

Aerobic training results in a substantial increase in energy expenditure, and thus provides particularly favorable conditions for the reduction of excess adipose tissue. Moreover, aerobic training prevents deterioration of the physical condition, and reduces morbidity and preterm mortality risk [33]. Beneficial effects of aerobic training can be observed primarily in the respiratory and musculoskeletal system [8, 11, 34].

The following should be considered during the selection of basic exercise forms: 1) activation of large muscle groups, 2) lack of breaks during the training, 3) the possibility to constantly monitor physical exercise intensity, 4) low risk of injury, and 5) acceptance of a given activity by the person exercising. General guidelines regarding aerobic exercises aimed at the reduction of body weight are listed in table I.

**Table I:** General guidelines regarding aerobic exercises aimed at body weight reduction. Tabela I. Ogólne zalecenia dotyczące ćwiczeń aerobowych ukierunkowanych na obniżenie masy ciała.

Aerobic exercises in body weight reduction *Ćwiczenia aerobowe w redukcji masy ciała*
1. Form: suitable for activation of large muscle groups and long-term maintenance of aerobic work *Forma: umożliwiająca aktywizację dużych grup mięśniowych i długotrwałe utrzymywanie pracy aerobowej*
2. Intensity: if not contraindicated otherwise, initially 40-60%, and then 60-70% of VOmax or HRR _2_*Intensywność: przy braku przeciwwskazań można na początku zalecać ćwiczenie przy 40-60%, a później przy 60-70% VOmax lub HRR _2_*
3. Frequency: whenever possible, initially 2-3 moderate-intensity training sessions per week, and then 1. a gradual increase to 4-5 or even 7 sessions weekly 1. *Częstotliwość ćwiczeń: jeżeli jest to możliwe poczynając od 2-3 umiarkowanych treningów w tygodniu,* 1. *następnie można ćwiczyć 4-5 razy, a nawet codziennie*
4. Duration of the training: determined by individual needs and objectives. Duration of aerobic activity should be gradually 1. and systematically increased, from 15-30 min to 60 min, but still at a recommended intensity level; 1. whenever possible and justified, the duration of exercises can be increased up to 300 min/week 1. *Czas trwania treningów: warunkowany jest osobniczo zróżnicowanymi potrzebami i celami. Czas systematycznie* 1. *i jednorazowo podejmowanej aerobowej pracy należy przedłużać, rozpoczynając od 15-30 do 60 minut* 1. *przy zachowaniu stosownej intensywności; jeżeli to możliwe i uzasadnione, czas ćwiczeń można zwiększać* 1. *do 300 minut/tydzień*
5. Duration of the program: depending on target body weight reduction and individual response to training 1. and dietary therapy. However, it should be remembered that physical exercises are a key determinant of health, 1. and thus, should never be abandoned at any point in a lifetime! 1. *Długotrwałość programu: poszczególne elementy programu zależą od pożądanych wielkości utraty masy ciała* 1. *i indywidualnie zróżnicowanej reaktywności na trening i dietoterapię. Należy jednak pamiętać, że na każdym etapie* 1. *życia ćwiczenia są niezbędnym warunkiem zdrowia – ćwiczyć należy systematycznie!*

Prepared based on: Heyward, Gibson [8]; Thompson [35].

The intensity of the training is typically determined and monitored based on the subject’s heart rate. Usually the measure of training intensity is the percentage of the maximal heart rate (HRmax) or the so-called heart rate reserve (HRR). In the case of training for persons with overweight or obesity, HRmax should be determined solely using empirically verified formulas, although they provide only rough estimates of these parameters. In recent years a widely-used formula for HRmax, i.e. HRmax = 220-age (in years) has been frequently replaced by a more adequate equation, usually:

HRmax = 208-(0.7 x age in years)

This approach is suitable for each subject, irrespective of age and sex [36]. Training for persons with overweight and obesity should also be adjusted to additional factors, such as age, co-existing ailments and comorbidities, medication history and general physical condition.

Based on the information about one’s degree of overweight or obesity, health status and other multiple determinants, an individual recommended zone of physical exercise intensity should be calculated using the formula below:

HRtr = HRs + %HRR

where:

HRtr – heart rate during training

HRs – heart rate at rest

HRR – heart rate reserve, i.e. the difference between maximal heart rate (HRmax) and heart rate at rest (HRs).

The intensity of training aimed at the maintenance or improvement of body composition should correspond to 40/50 - 85% of HRR [37].

Heart rate during training can be monitored continuously with a cardio monitor (pulsometer), usually placed on the chest or wrist. Most modern exercise ergometers (sport testers) are integrated with simple devices for easy control of physical exercise intensity.

A person participating in the training aimed at reduction of excess body weight should master the subjective assessment of exertion perceived during the exercise. Typically, this parameter is determined with the Borg Scale [38]. This scale proved to be useful for subjective assessment of exertion perceived during aerobic exercise; the scores, expressed using Borg points, were shown to strongly correlate with laboratory indices of exercise intensity.

Another simple method that can be used to assess individual response to physical exercise is conversation with the subject (also referred to as the talk test). The examiner observes the appearance of the subject, his/ her behavior, breathing depth and intensity, and coherence of responses. A subject who gives exhaustive responses to the examiner’s questions without pausing for breath, likely did not experience excessive exertion.

### Recommendations regarding resistance training

Aside from aerobic exercises, also resistance training is an integral component of the physical activity program. The aim of resistance training is to improve the biochemical and functional performance of specific skeletal muscles, to increase bone mineral density, and to induce favorable changes in the nervous system. Resistance training is not as effective in burning calories as aerobic exercises. However, it results in muscle mass gain, and thus improves insulin sensitivity and general immunity, facilitates the synthesis of albumins in the liver, and enhances the resting metabolic rate [8, 39].

The suitability of resistance training for the therapy of overweight and obesity is supported by the results of published studies in which this type of exercise contributed to the improvement of various health indices, quality of life and functional performance. Well-programmed resistance training may improve glucose tolerance, contribute to a decrease in excessively high diastolic and systolic blood pressure, reduce blood concentration of lipids, induce favorable changes in the structure and function of muscles, and improve bone condition. The beneficial effects of resistance training can be observed at a biochemical, functional and structural level [8, 40].

Resistance training for children and adolescents should follow the respective guidelines [8, 41]:

1. Strength training programs for pre-adolescents and adolescents can be safe and effective if proper resistance training techniques and safety precautions are followed.
2. Pre-adolescents and adolescents should avoid competitive weight lifting, power lifting, body building, and maximal lifts, until they reach physical and skeletal maturity.
3. specific strength training exercises should be learned initially with no load (resistance). Once the exercise skill has been mastered, incremental loads can be added.
4. It is better if resistance training is undertaken after general aerobic exercises; it should last no longer than 20-30 min. However, the importance of this type of training should never be underestimated. It is important to plan adequate breaks between individual exercises.
5. The persons exercising should gradually gain the ability to self-rate their perceived exertion (RPE). Usually, RPE should correspond to 12-13 points on the 20-point Borg Scale (i.e. to moderate exertion).
6. Consecutive resistance training sessions should be scheduled with at least 48-h intervals, to provide adequate time for regeneration of muscle tissue and general regeneration.
7. A general strengthening program should address all major muscle groups and exercises through the complete range of motion.
8. Any sign of injury or illness from strength training should be evaluated before continuing the exercise in question.
9. Each participant’s cognitive development, physical maturity, and training experience should be considered.
10. Each session should begin with a 5- to 10-min dynamic warm-up period.
11. Training should begin with 8-12 exercises that strengthen the upper body, lower body, and midsection.
12. It is recommended to initially perform 1 or 2 sets of 8-15 repetitions with a light to moderate load (about 60% 1 repetition maximum – RM) to learn the proper form and technique.
13. specific exercises that require balance and coordination should be included.
14. It is necessary to cool down with less intense activities and static stretching.

### High-intensity interval training (HIIT)

Based on the evidence from previous studies, it was generally assumed that persons with overweight and obesity should perform less intense exercises with relatively longer duration. High-intensity training with shorter duration was postulated to be too risky for the cardiovascular system, and to predispose to physiological exertion and injury. However, nowadays the strategy of HIIT is promoted as well, and thought to be an effective method for enhanced reduction of body fat [42, 43]. This form of training is considered a huge physical strain and a strong stimulator of biochemical and functional changes. Therefore, HIIT should be carefully implemented in body weight reduction programs and adjusted to the individual capabilities of the subjects.

HIIT programs include supramaximal, maximal or submaximal exercise followed by moderate aerobic exercise or restitution. However, the duration of the restitution break is too short for regeneration and normalization of vital functions. In its basic form HIIT is aimed at strengthening the cardiovascular system, improving functional performance, enhancing insulin sensitivity of the muscles and glucose metabolism. This form of training was also shown to be highly effective in the reduction of adipose tissue, BMI and waist circumference, improvement of body composition and sensitivity to insulin [44]. Also the physiological role of the so-called oxygen debt and enhanced post-exercise oxygen uptake has been emphasized as an important feature of HIIT. As a result, body fat is catabolized not only during the exercise but also up to 24 h thereafter. A post-exercise increase in the resting metabolic rate (even by several percent) results in enhanced catabolism of fatty acids. However, many issues related to the intensity and duration of HIIT, physiological mechanisms of adaptation and clinical application of this form of training still need to be addressed [27].

Sprint Interval Training (SIT) is a specific form of HIIT [44] comprising 5-15 cycles of exercise, each of which is 10-30 seconds, with very high intensity, corresponding to 90-95% of VO_2_max. Each cycle is followed by a short restitution period lasting no longer than 20-30 seconds. This type of exercise can be undertaken by young persons without any comorbidities. SIT was shown to exert favorable effects on multiple health indices: VO_2_max [45, 46], body composition [45], insulin resistance [47] and blood pressure [48]. The application of SIT as a component of various therapeutic programs is the subject of ongoing research [28]. Individual components of SIT, a form of HIIT, are listed in table II.

**Table II:** Individual components of high-intensity interval training (HIIT) aimed at the reduction of body weight, in the form of sprint interval training (SIT). Tabela II. Poszczególne elementy treningu interwałowego o wysokiej intensywności (HIIT) ukierunkowanego na redukcję masy ciała, w formule interwałowego treningu sprinterskiego (SIT).

Interval exercises in body weight reduction
1. Intensity: 90-95% of VOmax _2_
*Intensywność: 90-95% of VOmax _2_*
2. Duration of the interval exercise: 10-30 s
*Czas trwania wysiłku interwałowego: 10-30 s*
3. Duration of the restitution break: 20-30 s
*Czas wysiłku regenerującego: 20-30 s*
4. Number of repetitions (cycles): 5-15
*Liczba powtórzeń: 5-15*
5. Total duration of the interval exercises: 50-300 s
*Całkowity czas wysiłku interwałowego: 50-300 s*
6. Duration of the restitution break between repetitions (cycles): 60-180 s
*Czas przerwy między powtórzeniami: 60-180 s*
7. Total duration of the training: 15-25 min
*Całkowity czas trwania treningu: 15-25 minut*
8. Exercise forms: for example marching or running – on a treadmill or outdoors, exercise on a cycling ergometer,
climbing the stairs, running, marching at a fast pace, swimming, jump-rope workout.
Formy ćwiczeń: na przykład marsze lub bieganie – na bieżni mechanicznej lub w terenie, jazda na rowerze stacjonarnym,
*wchodzenie po schodach, bieg, szybki marsz, pływanie, podskoki z wykorzystaniem skakanki*.
9. Frequency: 2-3 times per week
*Częstotliwość: 2-3 razy w tygodniu*
10. Duration of the program: 3-6 months
*Czas programu: 3-6 miesięcy*

Training loads can be modified due to change in the proportion of time spent on intensive interval training and restitution time. In the case of beginners, this proportion should be 1:3. Then it can be changed to 1:2 in persons in a better physical condition, and to 1:1 or 1:0.5 in advanced subjects.

### Interventions based on physical exercises of various character in the therapy of overweight and obesity

Physical exercise-based interventions used to treat overweight and obesity in children and adolescents are usually conducted in a laboratory or clinical setting, or in the natural environment of home or school. Usually intervention programs include moderate- or high-intensity exercises with relatively short duration. Also the important role of resistance training applied alone or combined with other aerobic or anaerobic exercises has been emphasized by many authors [49-56].

Laframboise and deGraauw [57] reviewed published studies that analyzed the effectiveness of aerobic physical training in the reduction of body fat in school children and adolescents. Interventions lasting 8 months were shown to produce more benefits than those with a duration of up to 8 weeks. Authors of another review paper, Atlantis, Barnes and Singh [50], demonstrated that aerobic exercises with a duration of 155-180 min/ week are more effective in fat reduction than those lasting 120-150 min/week.

De Mello et al. [52] analyzed a group of 30 obese girls and boys aged 15 to 19 years, who participated in a one-year aerobic exercise programs (3 x 60 min per week). A significant reduction of fat mass was observed both after 6 months and at one year. However, even more favorable effects were noted when aerobic exercises were combined with resistance training. Similar results were also obtained by Damaso et al. [51] in a group of 116 adolescent girls and boys with obesity. However, also shorter programs may produce some beneficial effects. Wong et al. [58] analyzed the effects of a 12-week intervention (2 sessions per week) including various forms of outdoor and indoor activities in a group of obese adolescents. Exercise intensity corresponded to 65-85% of HRmax. The intervention resulted in improvement of several parameters, among them BMI, lean body mass and fat percentage. Also Alberga et al. [49] demonstrated that aerobic exercise, especially combined with resistance training, may improve the physical performance of obese adolescents.

Lee et al. [59] compared the effects of a 3-month physical activity program (180 min/week) based on aerobic or resistance exercises in a group of 12- to 18-year-old girls and boys with obesity. Although none of the study groups showed a reduction of body weight, the aerobic exercise program contributed to achieving a significant decrease in the visceral adipose tissue content. While individuals participating in the resistance training program showed a decrease in fat mass and visceral adipose tissue content, none of these changes were statistically significant.

Many previous studies analyzed the effects of isolated resistance training on the health indices in children and adolescents with overweight and obesity. According to Schranz and Tomkinson [55], resistance training may exert favorable effects on body composition, but the changes are small or very small. Instead, resistance training results in muscle mass gain.

Recently HIIT has been proposed as a low-volume alternative for more time-consuming aerobic training. The effects of HIIT have been a subject of many previous studies. Gibala et al. [60] demonstrated that interval exercises of short duration, with a total time of 2.5 h, stimulated similar changes in muscle biochemistry as conventional aerobic training lasting for 10.5 h. Boutcher [61] showed that HIIT resulted in the better utilization of fatty acids than typical moderate aerobic training, contributed to a marked increase in resting metabolic rate, decreased insulin resistance and improved the tolerance of glucose. This should probably be linked with the fact that HIIT involves approximately 80% of body muscles, as compared to only 40% during running at a moderate pace or riding a bike.

Fisher et al. [54] compared the effects of a 6-week training program with high and moderate intensity in a group of 17- to 22-year-old men with overweight or obesity. Both regimens resulted in a similar improvement of most cardiometabolic risk factors, and caused essentially the same reduction of body fat. Garcia-Hermoso et al. [62] conducted a meta-analysis of various physical activity programs for adolescents with obesity or overweight, and concluded that HITT contributes to a greater reduction of systolic blood pressure and more evident increase in VO_2_max than moderate-intensity continuous training, moderate-intensity interval training and low-intensity interval training.

Racil et al. [63] demonstrated that a 12-week HIIT program for 14-year-old girls with obesity produced more beneficial effects in terms of body fat and waist circumference reduction than a moderate-intensity interval training program of the same duration. Weston et al. [56] conducted an intervention among 14-year-old girls and boys with various body weight statuses. A 10-week HIIT program, including two weekly sessions held during curricular physical education classes and one additional extracurricular session per week, resulted in a reduction of waist circumference and plasma concentration of triglycerides and improved performance during a 20-m shuttle-run. However, no favorable changes were observed in body fat content. Buchan et al. [47] analyzed the effects of a 7-week training program (3 sessions per week) in a group of 16-year-olds subjected to either moderate-(420 min in total) or high-intensity (63 min) exercises. Individuals subjected to the moderate-intensity training showed an improvement of aerobic fitness, body fat percentage and BMI, whereas those subjected to the high-intensity exercises presented with lower systolic blood pressure and BMI, and better aerobic fitness.

The evidence from numerous previous studies analyzing the effectiveness of physical activity programs with moderate- or high-intensity exercises and resistance training [27, 28, 42-63] suggests that this type of intervention produces some health benefits also in terms of absolute and relative body composition. The protocol of the training should be adjusted to intervention objectives. Aerobic exercises are more effective in reducing body fat and improving body composition when combined with resistance training. HIIT produces similar or better results than moderate-intensity training and is more time-efficient.

## Conclusion

The health, social and economic consequences of the growing prevalence of overweight and obesity among children and adolescents can be devastating. Alarmingly, in a large proportion of subjects excess body weight observed at a younger age will also persist later in life. This justifies the implementation of effective preventive measures in various areas of health policy, as well as at the educational level. New health policies should be implemented primarily by physicians, nurses, personal trainers, physical education teachers and parents.

An important element during designing and implementing a body weight reduction program is participation in controlled forms of intense physical activity. The effects of participation in physical exercise-based programs are only seemingly small. In turn, the reduction of body weight solely with a restrictive diet has already been shown to be risky and less effective. Furthermore, a purely dietary approach may produce many unfavorable effects, such as a decrease in muscle mass, metabolic disorders, impaired immunity, loss of stamina, the yo-yo effect and hormonal disruption, e.g. enhanced release of cortisol. Depending on the form of training used in the therapy of overweight and obesity (HITT, moderate-intensity training, resistance training), its duration and intensity, the beneficial effects of the intervention may expand onto various functional and biochemical parameters of the body. Based on the results of different studies, HIIT combined with resistance training could be recommended as a suitable method for body weight reduction. It should be noted that the application of appropriate procedures for the overweight or obese person in reducing body weight is determined by individual needs and objectives and the individual response to exercise.
